# Supplementary material for: A map of single-phase high-entropy alloys
Source: Nat Commun. 2023 May 19;14:2856. doi: 10.1038/s41467-023-38423-7 (PMC10199023; doi:10.1038/s41467-023-38423-7)
Supplement: Supplementary file 1 — Supplementary Information [file 41467_2023_38423_MOESM1_ESM.pdf]

Supplementary Information
A map of single-phase high-entropy alloys

Wei Chen,[1] Antoine Hilhorst,[2] Georges Bokas,[1] Stéphane Gorsse,[3] Pascal J. Jacques,[2] and Geoffroy Hautier[1,4]

[1]*Institute of Condensed Matter and Nanoscicence (IMCN), Université catholique de Louvain, Chemin Etoiles 8, Louvain-la-Neuve 1348, Belgium*

[2]*Institute of Mechanics, Materials and Civil Engineering (IMAP), Place Sainte Barbe 2, Louvain-la-Neuve 1348, Belgium*

[3]*CNRS, University of Bordeaux, Bordeaux INP, ICMCB, UMR 5026, Pessac 33600, France*

[4]*Thayer School of Engineering, Dartmouth College, Hanover, New Hampshire 03755, USA*

(Dated: May 2, 2023)

LIST OF TABLES

1 Description of empirical rules and free-energy models . . . 2
2 Model validations . . . 2
3 Phase stability prediction of the 134 quaternary and quinary alloys . . . 3
4 Predicted structures of the 73 single-phase HEAs . . . 6
5 Statistics of phase stability for the 658,008 quinary alloys . . . 7

LIST OF FIGURES

1 Metal elements considered in the present study . . . 8
2 TPR vs FPR for the various models . . . 8
3 Formation enthalpy compared to the AFLOW data . . . 9
4 Predicted formation enthalpy pertinent to selected quinaries . . . 10
5 Intermetallics vs solid solution . . . 11
6 Intermetallics vs solid solution for selected quinaries . . . 12
7 Predicted formation enthalpy for Al–*X* and Zn–*X* solid solutions . . . 12
8 SEM and EDX of AlCoMnNiV . . . 13
9 SEM and EDX of CoFeMnNiZn . . . 14

Supplementary Table 1. Criteria for the formation of single-phase HEAs defined by various empirical rules (ERs) and free-energy models (FEMs).

ER1	$T_m \Delta S_{mix}/\lvert\Delta H_{mix}\rvert \geq 1.1, \delta < 0.066$[a]	Ref. 1
ER2	$-11.6 < \Delta H_{mix} < 3.2$ kJ/mol, $\delta < 0.066$	Ref. 2
ER3	$-11.6 < \Delta H_{mix} < 3.2$ kJ/mol, $\gamma < 1.175$[b]	Ref. 3
ER4	$\Delta S_{mix}/\delta^2 > 9.6$ kJ/mol/K	Ref. 4
FEM1	$-T_{ann}\Delta S_{mix} < \min(\Delta H^{IM}_{f,ij}) < 3.57$ meV/atom	Ref. 5
FEM2	$1 + 0.4 \times T_{ann}\Delta S_{mix}/\lvert\Delta H_{mix}\rvert < \Delta H^{IM}_{f}/\Delta H_{mix}$	Ref. 6
VEC	FCC if VEC ≥ 8, BCC if VEC ≤ 6.87, mixed phase otherwise	Ref. 7

[a] $\delta = \sqrt{\sum_i c_i \left(1 - \frac{r_i}{\bar{r}}\right)^2}$, where c_i is the atomic percentage of the ith component, r_i the atomic radius and $\bar{r} = \sum_i c_i r_i$.

[b] $\gamma = \frac{\omega_s}{\omega_l}$, where $\omega_{s,l} = 1 - \sqrt{\frac{(r_{s,l}+\bar{r})^2 - \bar{r}^2}{(r_{s,l}+\bar{r})^2}}$. $r_{s,l}$ refers to the radius of the smallest and the largest atoms, respectively.

Supplementary Table 2. Model validations for the present and the two free-energy models as a function of temperature (in K). The true positive rate (TPR) refers to the percentage of the single-phase solid solutions predicted from a pool of 73 experimentally known single-phase quaternary and quinary HEAs. The false positive rate (FPR) refers to the percentage of the single-phase solid solutions predicted from a pool of 61 multi-phase quaternary and quinary alloys. The overall accuracy is defined as $[\text{TPR} \times 73 + (100 - \text{FPR}) \times 61]/134$.

	Present			FEM1			FEM2		
T_a (K)	TPR	FPR	Acc.	TPR	FPR	Acc.	TPR	FPR	Acc.
800	22	2	57	22	11	52	42	36	52
850	26	3	58	26	11	54	42	39	51
900	32	7	60	29	15	54	44	39	51
950	37	10	61	32	18	54	47	43	51
1000	44	10	65	37	21	56	47	44	51
1050	45	10	66	37	21	56	47	46	50
1100	47	11	66	41	26	56	47	46	50
1150	47	20	62	41	26	56	47	48	49
1200	55	20	66	42	28	56	49	48	51
1250	60	20	69	44	30	56	49	48	51
1300	63	20	71	47	30	57	52	48	52
1350	70	21	74	47	30	57	58	48	55
1400	74	28	73	49	31	58	59	51	54
1450	75	30	73	49	31	58	62	52	55
1500	79	38	72	58	33	62	62	54	54
1550	82	43	71	58	33	62	63	56	54
1600	88	46	72	58	33	62	64	56	55

Supplementary Table 3: Predicted phases of the 134 experimentally confirmed equimolar quaternary and quinary alloys (compiled from Refs. 8–10) by the present approach along with the existing empirical rules (ERs) and free-energy models (FEMs). The temperature for the present and the two FEMs (FEM1 and FEM2) is set to 1350, 1500, and 1350 K, respectively. The enthalpy of mixing ($\Delta H^{\mathrm{M}}_{\mathrm{mix}}$, in meV/atom) refers to the binary terms taken from Ref. 11 on the basis of Miedema's scheme [12]. The enthalpy of formation for the intermetallics ($\Delta H^{\mathrm{IM}}_{\mathrm{f}}$, in meV/atom) is defined by $\Delta H^{\mathrm{IM}}_{\mathrm{f}} = 4\sum_{j>i} c_i c_j \Delta H^{\mathrm{IM}}_{ij}$ where the binary term $\Delta H^{\mathrm{IM}}_{ij}$ is obtained with DFT calculations. The definitions of the ERs and FEMs are given in Table 1. Single phase and multi phase are denoted by the solid (■) and the open (◎) markers, respectively. Correct (incorrect) predictions are highlighted in blue (red).

Alloy	T_m(K)	$\Delta H^{\mathrm{M}}_{\mathrm{mix}}$	$\Delta H^{\mathrm{IM}}_{\mathrm{f}}$	Expt.	Present	ER1	ER2	ER3	ER4	FEM1	FEM2
AlCoCrNi	1652	−162	−365	◎	◎	■	◎	◎	◎	◎	◎
AlCoFeNi	1560	−141	−435	■	◎	■	◎	◎	◎	◎	◎
AlCoNiTi	1593	−346	−654	◎	◎	◎	◎	◎	◎	◎	◎
AlCrCuFe	1571	−12	−109	◎	◎	■	■	■	◎	◎	◎
AlCrFeNi	1663	−138	−294	■	◎	■	◎	◎	◎	◎	◎
AlCrMoNb	2190	−118	−258	◎	◎	■	■	■	◎	◎	◎
AlCrMoTi	1988	−143	−289	■	■	■	◎	◎	◎	◎	◎
AlCuNiTi	1490	−257	−483	■	◎	◎	◎	◎	◎	◎	◎
AlFeNiTi	1603	−300	−593	◎	◎	◎	◎	◎	◎	◎	◎
AlHfNbTi	2033	−208	−322	◎	■	◎	◎	◎	◎	◎	◎
AlHfTaTi	2168	−214	−282	◎	■	◎	◎	◎	◎	◎	◎
AlMoNbTi	2130	−156	−369	■	■	■	◎	◎	◎	◎	◎
AlNbTaTi	2229	−165	−287	■	■	■	◎	◎	◎	◎	◎
AlNbTiV	1952	−168	−291	■	■	■	◎	◎	◎	◎	◎
AlNbTiZr	1938	−222	−339	◎	■	◎	◎	◎	◎	◎	◎
CoCrCuFe	1779	65	49	■	◎	■	◎	◎	■	■	■
CoCrFeNi	1872	−39	−45	■	■	■	■	■	■	■	■
CoCrMnNi	1799	−59	−58	■	■	■	■	■	■	■	■
CoCrMoNb	2398	−120	−80	◎	◎	■	◎	◎	■	■	■
CoCuFeNi	1666	53	−17	■	■	■	◎	◎	■	■	■
CoFeMnNi	1706	−40	−100	■	■	■	■	■	■	■	■
CoFeNiPd	1784	−22	−79	■	◎	■	■	■	■	■	■
CoFeNiV	1872	−108	−198	■	■	■	■	■	■	◎	◎
CoFeReRu	2411	−13	−47	■	◎	■	■	■	■	■	■
CoNiRhRu	2085	−6	53	■	◎	■	■	■	■	■	■
CrFeMnNi	1810	−41	−64	■	■	■	■	■	■	■	■
CrMoTaTi	2577	−54	−135	◎	◎	■	■	■	■	◎	◎
CrNbTaTi	2540	−47	−64	◎	◎	■	■	■	■	■	■
CrNbTiW	2642	−67	−70	◎	◎	■	■	■	■	■	■
CrNbTiZr	2250	−54	−39	◎	◎	■	■	■	◎	■	■
CrTaTiV	2398	−45	−94	◎	■	■	■	■	■	■	■
CrTaVW	2837	−44	−129	◎	◎	■	■	■	■	■	◎
HfNbTaTi	2622	27	24	■	■	■	■	■	◎	■	■
HfNbTaZr	2668	34	26	■	■	■	◎	◎	■	■	■
HfNbTiZr	2331	25	9	■	■	■	■	■	◎	■	■
HfScTiZr	2097	43	−8	◎	■	■	◎	◎	◎	■	■
HfTaTiZr	2466	18	25	■	■	■	■	■	◎	■	■
HfTiYZr	2094	92	48	◎	◎	◎	◎	◎	◎	■	■
MoNbTaTi	2719	−28	−113	■	■	■	■	■	■	◎	◎
MoNbTaV	2780	−33	−156	■	■	■	■	■	■	◎	◎
MoNbTaW	3158	−69	−131	■	■	■	■	■	■	◎	■
MoNbTiV	2442	−26	−106	■	■	■	■	■	■	■	◎
MoNbTiZr	2429	−25	−93	■	■	■	■	■	◎	■	◎
MoPdRhRu	2392	−90	−99	■	◎	■	■	■	◎	◎	■
MoTaTiV	2578	−25	−135	■	■	■	■	■	■	◎	◎
NbTaTiV	2541	−1	−25	■	■	■	■	■	■	■	■
NbTaTiW	2919	−47	−58	■	■	■	■	■	■	■	■
NbTaTiZr	2527	25	28	■	■	■	■	■	◎	■	■
NbTaVW	2980	−48	−112	■	■	■	■	■	◎	■	■

Continued on next page

Supplementary Table 3 – *Continued from previous page*

System	T_m(K)	$\Delta H_{\mathrm{mix}}^{\mathrm{M}}$	$\Delta H_{\mathrm{f}}^{\mathrm{IM}}$	Expt.	Present	ER1	ER2	ER3	ER4	FEM1	FEM2
NbTiVZr	2250	−2	16	■	blue ■	blue ■	blue ■	blue ■	red ⊙	blue ■	blue ■
NiPdPtRh	1959	−9	−34	■	blue ■	blue ■	blue ■	blue ■	blue ■	blue ■	blue ■
AgAuCuPdPt	1560	−65	−99	■	blue ■	red ⊙	red ⊙	red ⊙	red ⊙	blue ■	blue ■
AlCoCrCuFe	1610	−37	−169	⊙	blue ⊙	red ■	red ■	red ■	blue ⊙	blue ⊙	blue ⊙
AlCoCrFeNi	1684	−128	−300	■	red ⊙	blue ■	red ⊙	red ⊙	red ⊙	red ⊙	red ⊙
AlCoCuFeNi	1520	−65	−294	⊙	blue ⊙	red ■	red ■	red ■	red ■	blue ⊙	blue ⊙
AlCoCuNiZn	1296	−93	−294	⊙	blue ⊙	red ■	red ■	red ■	red ■	blue ⊙	blue ⊙
AlCoFeNiTi	1636	−271	−557	■	red ⊙	red ⊙	red ⊙	red ⊙	red ⊙	red ⊙	red ⊙
AlCrCuFeMg	1441	50	−57	⊙	blue ⊙	red ■	blue ⊙	blue ⊙	blue ⊙	blue ⊙	red ■
AlCrCuFeMn	1560	−29	−119	⊙	blue ⊙	red ■	red ■	red ■	blue ⊙	blue ⊙	blue ⊙
AlCrCuFeNi	1602	−53	−194	⊙	blue ⊙	red ■	red ■	red ■	blue ⊙	blue ⊙	blue ⊙
AlCrFeMoNi	1910	−111	−251	⊙	blue ⊙	red ■	red ■	red ■	blue ⊙	blue ⊙	blue ⊙
AlCrMoNbTi	2140	−140	−279	⊙	blue ⊙	red ■	blue ⊙	blue ⊙	blue ⊙	blue ⊙	blue ⊙
AlCrMoSiTi	1927	−241	−419	⊙	blue ⊙	blue ⊙	blue ⊙	blue ⊙	blue ⊙	blue ⊙	blue ⊙
AlCrMoTiW	2329	−103	−223	■	blue ■	blue ■	blue ■	blue ■	red ⊙	red ⊙	red ⊙
AlCrNbTiV	1997	−151	−249	⊙	red ■	red ■	blue ⊙	blue ⊙	blue ⊙	blue ⊙	blue ⊙
AlCuFeNiTi	1554	−192	−425	■	red ⊙	blue ■	red ⊙	red ⊙	red ⊙	red ⊙	red ⊙
AlCuMnNiPt	1516	−231	−479	■	red ⊙	red ⊙	red ⊙	red ⊙	blue ■	red ⊙	red ⊙
AlCuTaVW	2292	−41	−181	⊙	blue ⊙	red ■	red ■	red ■	blue ⊙	blue ⊙	blue ⊙
AlCuTiYZr	1632	−245	−339	⊙	blue ⊙	blue ⊙	blue ⊙	blue ⊙	blue ⊙	blue ⊙	blue ⊙
AlMoNbTiV	2141	−131	−306	■	blue ■	blue ■	red ⊙	red ⊙	red ⊙	red ⊙	red ⊙
AlMoTaTiV	2249	−132	−307	■	blue ■	blue ■	red ⊙	red ⊙	red ⊙	red ⊙	red ⊙
AlNbTaTiV	2219	−138	−251	■	blue ■	blue ■	red ⊙	red ⊙	red ⊙	red ⊙	red ⊙
AlNbTaTiZr	2208	−167	−257	⊙	red ■	blue ⊙	blue ⊙	blue ⊙	blue ⊙	blue ⊙	blue ⊙
AlNbTiVZr	1987	−180	−260	⊙	red ■	blue ⊙	blue ⊙	blue ⊙	blue ⊙	blue ⊙	blue ⊙
AuCuPdPtSn	1414	−162	−309	■	red ⊙	blue ■	red ⊙	red ⊙	blue ■	red ⊙	red ⊙
CoCrCuFeNi	1769	34	8	⊙	blue ⊙	red ■	blue ⊙	blue ⊙	red ■	red ■	red ■
CoCrCuNiZn	1545	−3	−44	■	red ⊙	blue ■	blue ■	blue ■	blue ■	red ⊙	blue ■
CoCrFeHfNi	1999	−202	−254	⊙	blue ⊙	red ■	blue ⊙	blue ⊙	blue ⊙	blue ⊙	red ■
CoCrFeMnNi	1801	−43	−64	■	blue ■	blue ■	blue ■	blue ■	blue ■	blue ■	blue ■
CoCrFeMoNi	2077	−48	−45	⊙	blue ⊙	red ■	red ■	red ■	red ■	red ■	red ■
CoCrFeNbNi	2047	−153	−131	⊙	blue ⊙	red ■	blue ⊙	blue ⊙	red ■	blue ⊙	red ■
CoCrFeNiPd	1863	−59	−62	■	blue ■	blue ■	blue ■	blue ■	blue ■	blue ■	blue ■
CoCrFeNiTa	2155	−149	−180	⊙	blue ⊙	red ■	blue ⊙	blue ⊙	red ■	blue ⊙	red ■
CoCrFeNiTi	1886	−169	−258	■	red ⊙	blue ■	red ⊙	red ⊙	blue ■	red ⊙	red ⊙
CoCrFeNiV	1934	−93	−139	⊙	red ■	red ■	red ■	red ■	red ■	blue ⊙	red ■
CoCrFeNiW	2236	−31	−59	⊙	blue ⊙	red ■	red ■	red ■	red ■	red ■	red ■
CoCrFeNiY	1857	−95	−116	⊙	blue ⊙	blue ⊙	blue ⊙	blue ⊙	blue ⊙	blue ⊙	red ■
CoCrFeNiZr	1923	−233	−206	⊙	blue ⊙	red ■	blue ⊙	blue ⊙	blue ⊙	blue ⊙	red ■
CoCrMnNiV	1876	−95	−170	⊙	red ■	red ■	red ■	red ■	red ■	blue ⊙	blue ⊙
CoCrMoNbTi	2307	−139	−160	⊙	blue ⊙	red ■	blue ⊙	blue ⊙	red ■	blue ⊙	red ■
CoCrNiTiV	1960	−195	−245	⊙	blue ⊙	red ■	blue ⊙	blue ⊙	red ■	blue ⊙	red ■
CoCuFeMnNi	1637	18	−42	■	red ⊙	blue ■	blue ■	blue ■	blue ■	blue ■	blue ■
CoCuFeMoNi	1912	41	−21	■	red ⊙	blue ■	red ⊙	red ⊙	blue ■	blue ■	blue ■
CoCuFeNiPd	1699	2	−50	■	red ⊙	blue ■	blue ■	blue ■	blue ■	blue ■	blue ■
CoCuFeNiPt	1741	−27	−112	■	blue ■	blue ■	blue ■	blue ■	blue ■	red ⊙	red ⊙
CoCuFeNiRu	1854	38	21	■	blue ■	blue ■	red ⊙	red ⊙	blue ■	blue ■	blue ■
CoCuFeNiTi	1721	−113	−243	■	red ⊙	blue ■	blue ■	blue ■	blue ■	red ⊙	red ⊙
CoCuNiPdPt	1745	−45	−88	■	red ⊙	blue ■	blue ■	blue ■	blue ■	blue ■	blue ■
CoFeIrPdPt	2037	−51	−99	■	blue ■	blue ■	blue ■	blue ■	blue ■	red ⊙	blue ■
CoFeMnMoNi	1944	−41	−103	⊙	blue ⊙	red ■	red ■	red ■	red ■	red ■	red ■
CoFeMnNiTi	1753	−171	−324	⊙	blue ⊙	red ■	blue ⊙	blue ⊙	red ■	blue ⊙	blue ⊙
CoFeMnNiV	1802	−92	−211	⊙	red ■	red ■	red ■	red ■	red ■	blue ⊙	blue ⊙
CoFeMoNiV	2077	−92	−170	⊙	red ■	red ■	red ■	red ■	red ■	blue ⊙	blue ⊙
CoFeNiPdPt	1835	−52	−131	■	blue ■	blue ■	blue ■	blue ■	blue ■	red ⊙	red ⊙
CoIrNiRhRu	2216	−12	11	■	blue ■	blue ■	blue ■	blue ■	blue ■	blue ■	blue ■
CrCuFeMnNi	1719	28	−9	⊙	blue ⊙	red ■	red ■	red ■	red ■	red ■	red ■
CrCuFeMoNi	1995	48	17	■	red ⊙	blue ■	red ⊙	red ⊙	blue ■	blue ■	blue ■
CrFeMnNiTi	1836	−137	−255	⊙	blue ⊙	red ■	blue ⊙	blue ⊙	red ■	blue ⊙	blue ⊙
CrFeMoNbTi	2316	−96	−150	⊙	blue ⊙	red ■	red ■	red ■	red ■	blue ⊙	red ■

Continued on next page

Supplementary Table 3 – *Continued from previous page*

System	T_m(K)	$\Delta H_{\mathrm{mix}}^{\mathrm{M}}$	$\Delta H_{\mathrm{f}}^{\mathrm{IM}}$	Expt.	Present	ER1	ER2	ER3	ER4	FEM1	FEM2
CrFeMoNbV	2364	−69	−108	◎	◎	■	■	■	■	■	■
CrHfNbTiZr	2301	−44	−46	◎	◎	■	■	■	◎	■	■
CrMoNbTiW	2692	−58	−86	◎	◎	■	■	■	■	■	■
CrNbTiVZr	2236	−49	−37	◎	◎	■	■	■	◎	■	■
CuFeMnNiPt	1691	−79	−170	◎	■	■	■	■	■	◎	◎
FeMoNbTiV	2316	−86	−185	◎	◎	■	■	■	■	◎	◎
HfMoNbTaTi	2677	−13	−90	■	■	■	■	■	■	■	◎
HfMoNbTaZr	2714	−13	−84	■	■	■	■	■	■	■	■
HfMoNbTiZr	2444	−16	−89	■	■	■	■	■	◎	■	■
HfMoTaTiZr	2552	−20	−92	■	■	■	■	■	◎	■	■
HfNbTaTiZr	2523	28	24	■	■	■	■	■	◎	■	■
HfNbTiVZr	2302	2	10	◎	◎	■	■	■	◎	■	■
HfScTiYZr	2038	88	31	◎	◎	◎	◎	◎	◎	■	■
IrOsReRhRu	2870	−3	−134	■	■	■	■	■	■	◎	◎
IrPdPtRhRu	2290	25	−23	■	■	■	■	■	■	■	■
MoNbReTaW	3218	−145	−196	■	■	■	◎	◎	■	◎	■
MoNbTaTiV	2612	−24	−114	■	■	■	■	■	■	■	◎
MoNbTaTiW	2914	−54	−118	■	■	■	■	■	■	■	■
MoNbTaVW	2963	−49	−146	■	■	■	■	■	■	■	◎
MoNbTiVZr	2380	−27	−78	■	◎	■	■	■	◎	■	■
MoTaVWZr	2838	−51	−143	◎	◎	■	■	■	◎	■	◎
NbNiTaTiW	2681	−190	−237	◎	◎	■	◎	◎	■	◎	■
NbReTaTiV	2725	−146	−253	■	■	■	◎	◎	■	◎	◎
NbSnTaTiZr	2123	−97	−183	◎	◎	■	■	■	■	◎	◎
NbTaTiVW	2772	−37	−73	■	■	■	■	■	■	■	■

Supplementary Table 4. Structures of the 73 experimentally confirmed single-phase HEAs predicted by the present and the VEC model.

Single-phase HEA	Expt.	Present	VEC
AlCoFeNi	BCC	BCC	IM
AlCrFeNi	BCC	BCC	BCC
AlCrMoTi	BCC	BCC	BCC
AlCuNiTi	FCC	BCC	IM
AlMoNbTi	BCC	BCC	BCC
AlNbTaTi	BCC	BCC	BCC
AlNbTiV	BCC	BCC	BCC
CoCrCuFe	FCC	FCC	FCC
CoCrFeNi	FCC	FCC	FCC
CoCrMnNi	FCC	HCP	IM
CoCuFeNi	FCC	FCC	FCC
CoFeMnNi	FCC	FCC	FCC
CoFeNiPd	FCC	BCC	FCC
CoFeNiV	FCC	BCC	IM
CoFeReRu	HCP	BCC	IM
CoNiRhRu	FCC	HCP	FCC
CrFeMnNi	FCC	FCC	IM
HfNbTaTi	BCC	BCC	BCC
HfNbTaZr	BCC	BCC	BCC
HfNbTiZr	BCC	BCC	BCC
HfTaTiZr	BCC	HCP	BCC
MoNbTaTi	BCC	BCC	BCC
MoNbTaV	BCC	BCC	BCC
MoNbTaW	BCC	BCC	BCC
MoNbTiV	BCC	BCC	BCC
MoNbTiZr	BCC	BCC	BCC
MoPdRhRu	HCP	FCC	FCC
MoTaTiV	BCC	BCC	BCC
NbTaTiV	BCC	BCC	BCC
NbTaTiW	BCC	BCC	BCC
NbTaTiZr	BCC	BCC	BCC
NbTaVW	BCC	BCC	BCC
NbTiVZr	BCC	BCC	BCC
NiPdPtRh	FCC	FCC	FCC
AgAuCuPdPt	FCC	FCC	FCC
AlCoCrFeNi	BCC	HCP	IM
AlCoFeNiTi	BCC	BCC	BCC
AlCrMoTiW	BCC	BCC	BCC
AlCuFeNiTi	FCC	BCC	IM
AlCuMnNiPt	FCC	HCP	FCC
AlMoNbTiV	BCC	BCC	BCC
AlMoTaTiV	BCC	BCC	BCC
AlNbTaTiV	BCC	BCC	BCC
AuCuPdPtSn	FCC	HCP	FCC
CoCrCuNiZn	FCC	FCC	FCC
CoCrFeMnNi	FCC	FCC	IM
CoCrFeNiPd	FCC	FCC	FCC
CoCrFeNiTi	FCC	BCC	IM
CoCuFeMnNi	FCC	FCC	FCC
CoCuFeMoNi	FCC	FCC	FCC
CoCuFeNiPd	BCC	FCC	FCC
CoCuFeNiPt	FCC	FCC	FCC
CoCuFeNiRu	HCP	BCC	FCC
CoCuFeNiTi	FCC	BCC	FCC
CoCuNiPdPt	FCC	FCC	FCC
CoFeIrPdPt	FCC	FCC	FCC
CoFeNiPdPt	FCC	FCC	FCC
CoIrNiRhRu	FCC	HCP	FCC
CrCuFeMoNi	FCC	FCC	FCC
HfMoNbTaTi	BCC	BCC	BCC
HfMoNbTaZr	BCC	BCC	BCC
HfMoNbTiZr	BCC	BCC	BCC
HfMoTaTiZr	BCC	BCC	BCC
HfNbTaTiZr	BCC	BCC	BCC
IrOsReRhRu	HCP	HCP	FCC
IrPdPtRhRu	FCC	FCC	FCC
MoNbReTaW	BCC	BCC	BCC
MoNbTaTiV	BCC	BCC	BCC
MoNbTaTiW	BCC	BCC	BCC
MoNbTaVW	BCC	BCC	BCC
MoNbTiVZr	BCC	BCC	BCC
NbReTaTiV	BCC	BCC	BCC
NbTaTiVW	BCC	BCC	BCC
Accuracy (%)		74	78

Supplementary Table 5. Number (N) of quinaries that are predicted as stable single-phase solid solutions at $T_a = 0.9T_m$ and 1350 K from a total of 658,008 candidates. The percentage (pct) with respect to the numer of candidates is also given. The refractory compounds refer to the quinaries comprising at least one refractory element.

	Total	FCC	HCP	BCC	Refractory
$T_a = 0.9T_m$					
N	30,201	1664	5906	22,631	23,781
pct (%)	4.6	0.3	0.9	3.4	3.6
$T_a = 1350$ K					
N	4512	306	704	3502	3722
pct (%)	0.7	0.0	0.1	0.5	0.6

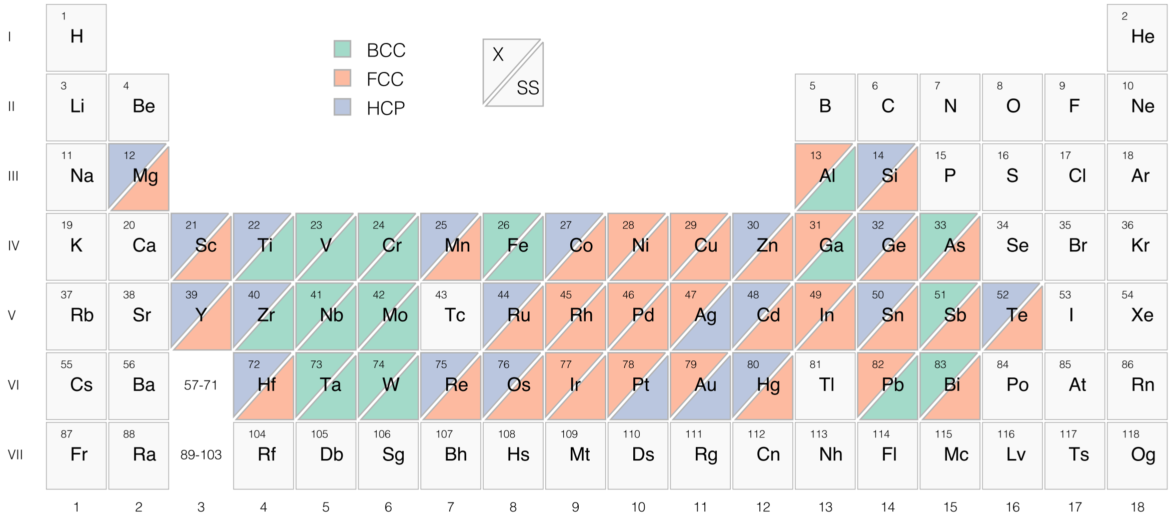

Supplementary Fig. 1. Metal elements considered in the present study. The ground-state elemental phase at 0 K is shown by the upper triangle, whereas the most stable phase of the element found in its binary solid solutions (SS) is shown by the lower triangle.

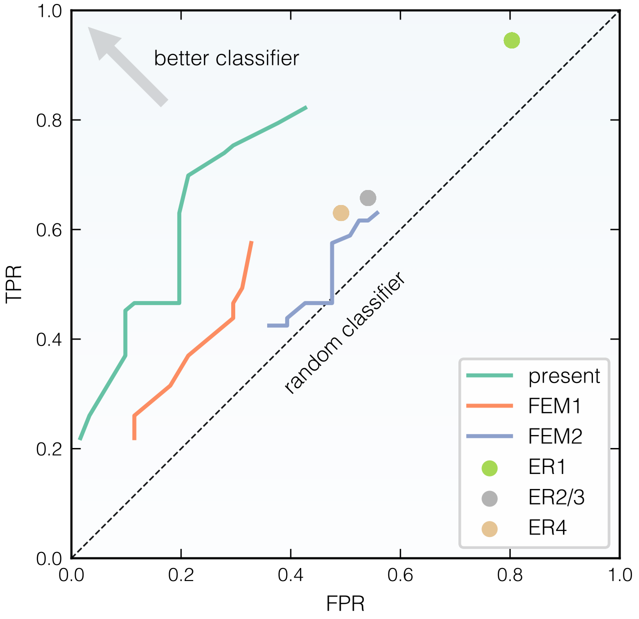

Supplementary Fig. 2. True positive ratio (TPR) vs false positive ratio (FPR) for the present and the previously developed models. Except for the ERs, the TRP and FPR are assessed for temperatures ranging from 800 to 1600 K. Analogous to the receiver operating characteristic (ROC) analysis, models exhibiting its presence closer to the upper left corner are better at classifying single-phase HEAs and multi-phase alloys.

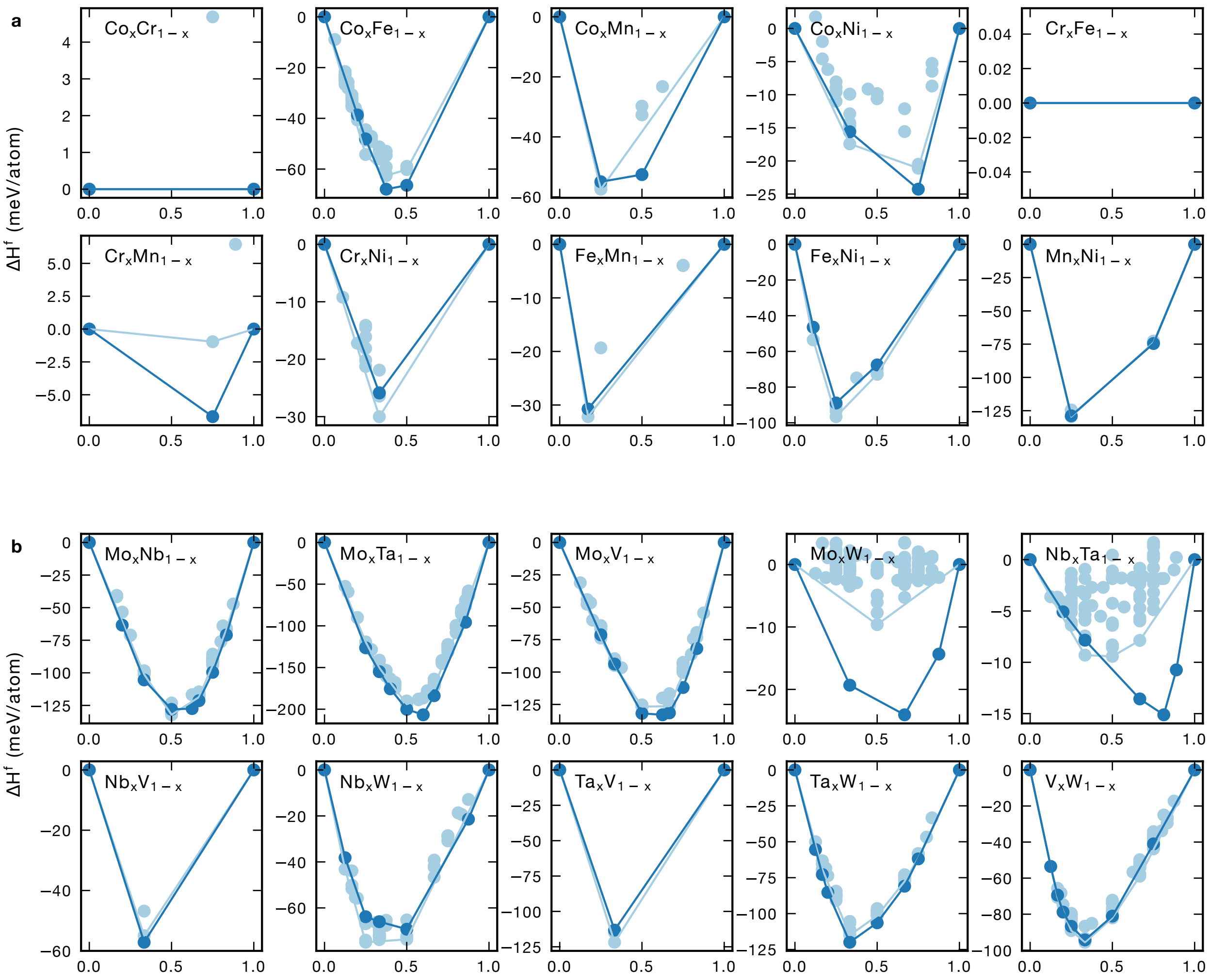

Supplementary Fig. 3. Predicted formation enthalpy vs atomic fraction for the binary intermetallic subsystems of (**a**) the Cantor alloy (CoCrFeMnNi) and (**b**) the refractory alloy (MoNbTaVW). The AFLOW LIB2 dataset and the present results are shown by the light and dark blue markers, respectively. The convex hulls are indicated. For the AFLOW dataset an energy above hull cutoff of 10 meV/atom is applied. The present results contain only the stable compounds.

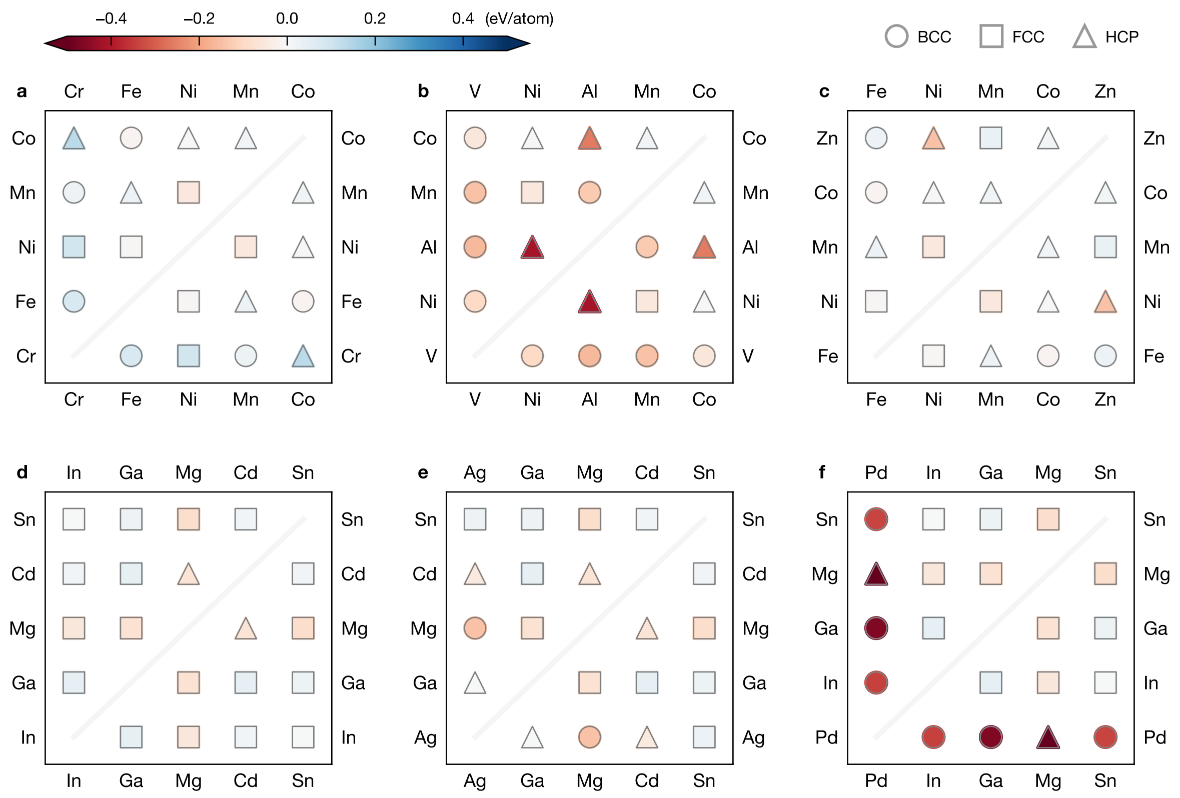

Supplementary Fig. 4. Predicted formation enthalpy of binary solid solutions for the constituent elements of (**a**) CoCrFeMnNi, (**b**) AlCoMnNiV, (**c**) CoFeMnNiZn, (**d**) CdGaInMgSn, (**e**) AgCdGaMgSn, and (**f**) GaInMgPdSn.

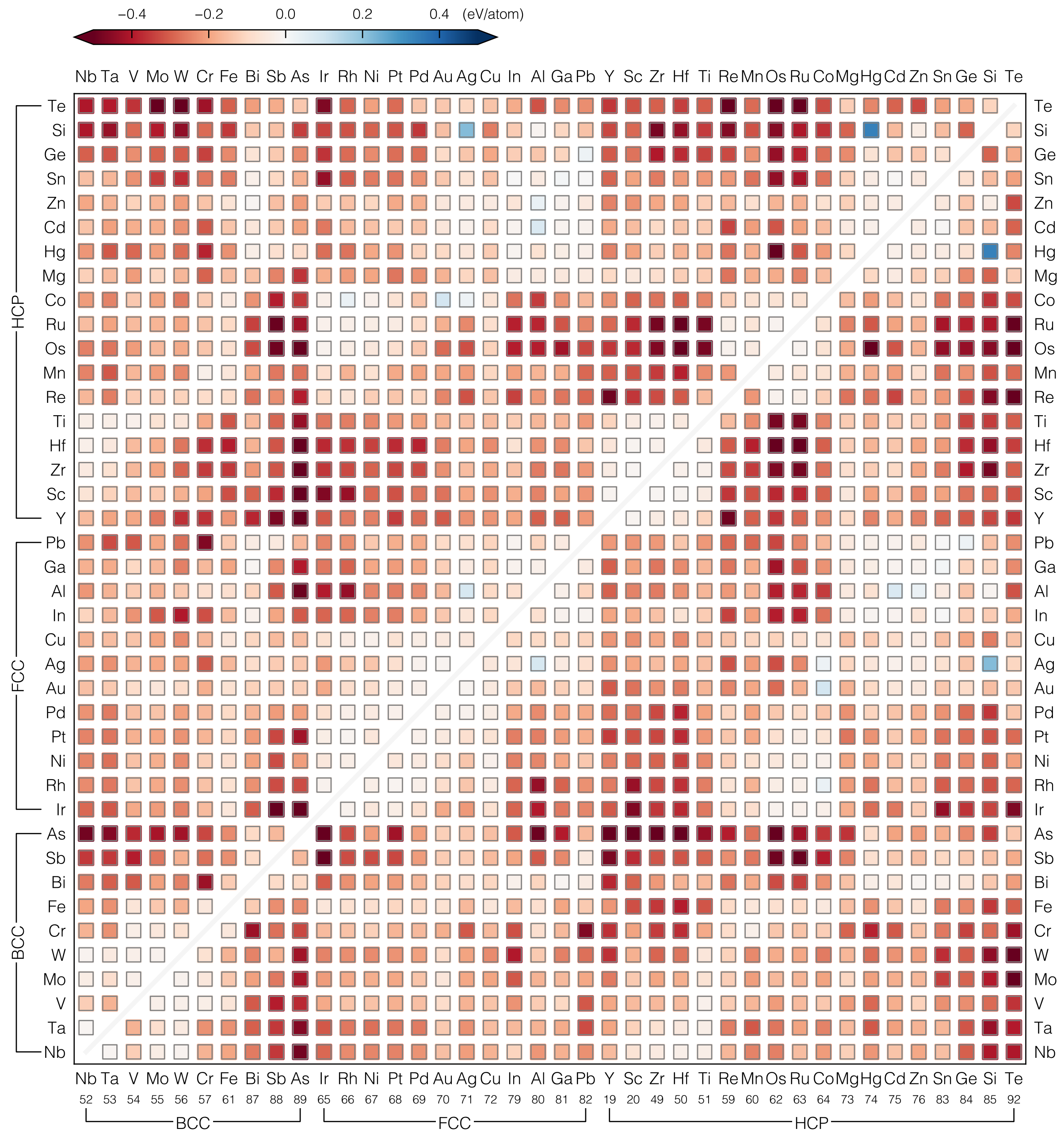

Supplementary Fig. 5. Predicted formation enthalpy of the binary intermetallics relative to that of the solid solution. Darker red suggests the presence of stronger competing intermetallic phases for a given binary system.

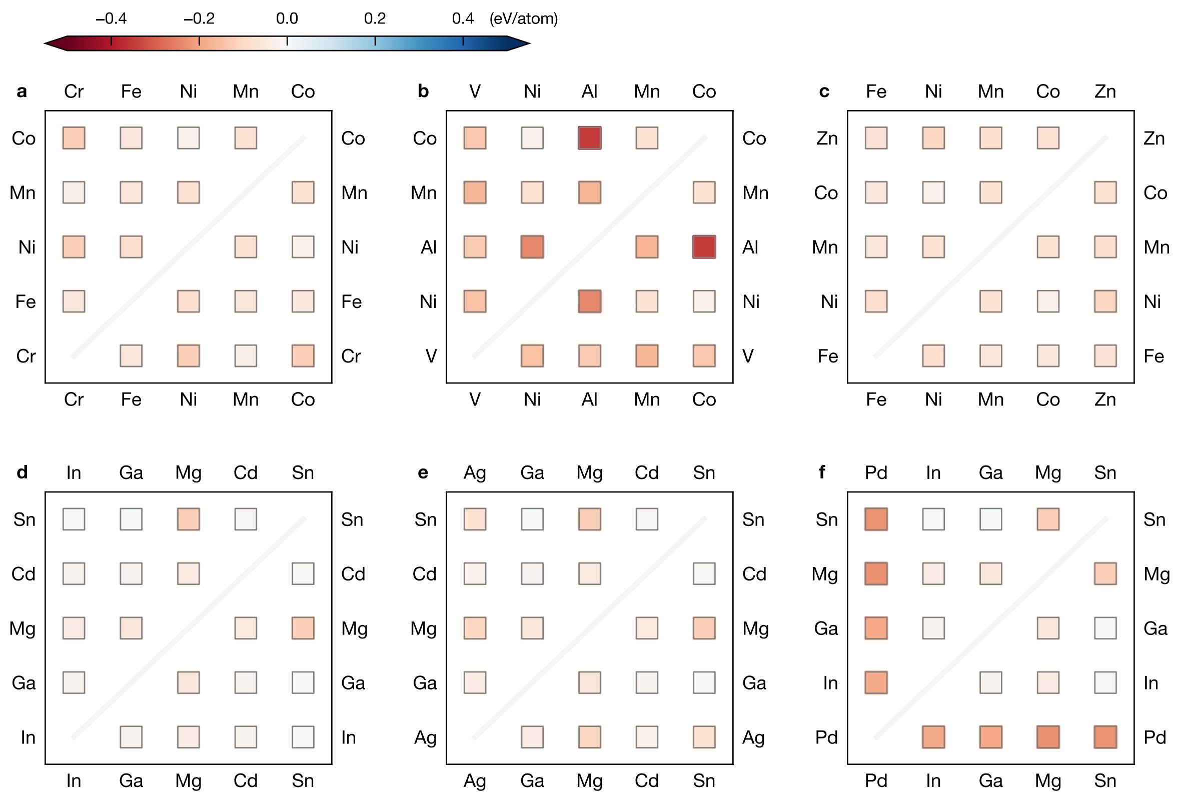

Supplementary Fig. 6. Same as Fig. 5 but only for the elements of (**a**) CoCrFeMnNi, (**b**) AlCoMnNiV, (**c**) CoFeMnNiZn, (**d**) CdGaInMgSn, (**e**) AgCdGaMgSn, and (**f**) GaInMgPdSn.

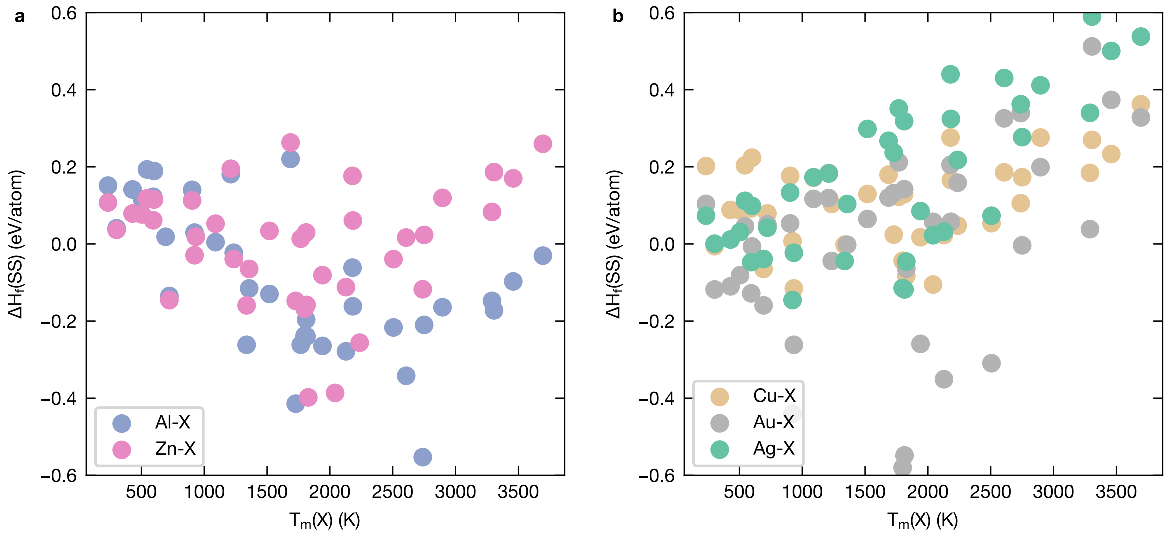

Supplementary Fig. 7. **a** Predicted formation enthalpy for Al–X and Zn–X solid solutions as a function of the melting point of element X. **b** is for Cu–X, Au–X, and Ag–X.

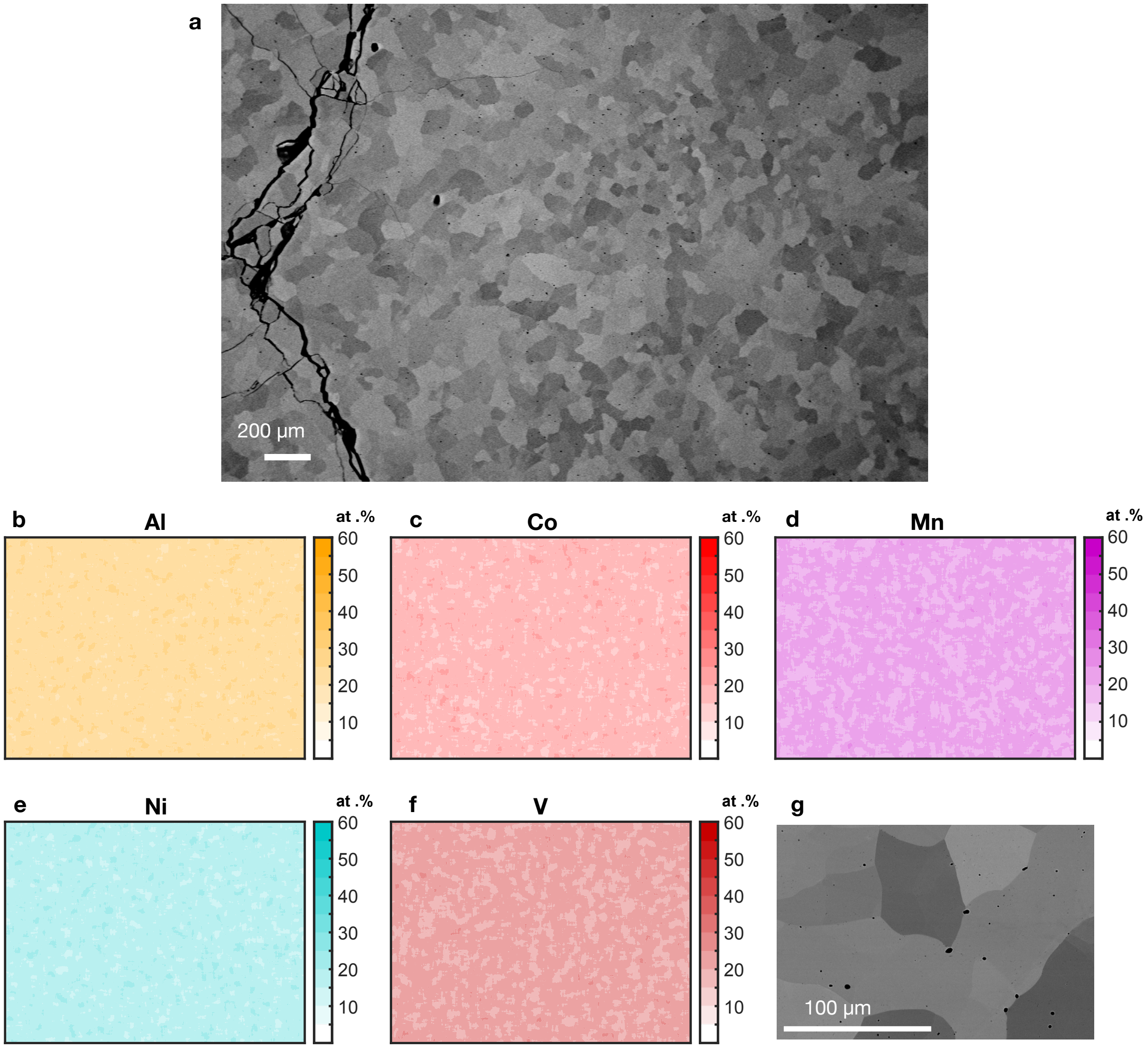

Supplementary Fig. 8. **a** Low magnification SEM micrograph of AlCoMnNiV taken under secondary-electrons (SE) mode. **b-f** EDX mapping of the BCC matrix of the region **g**.

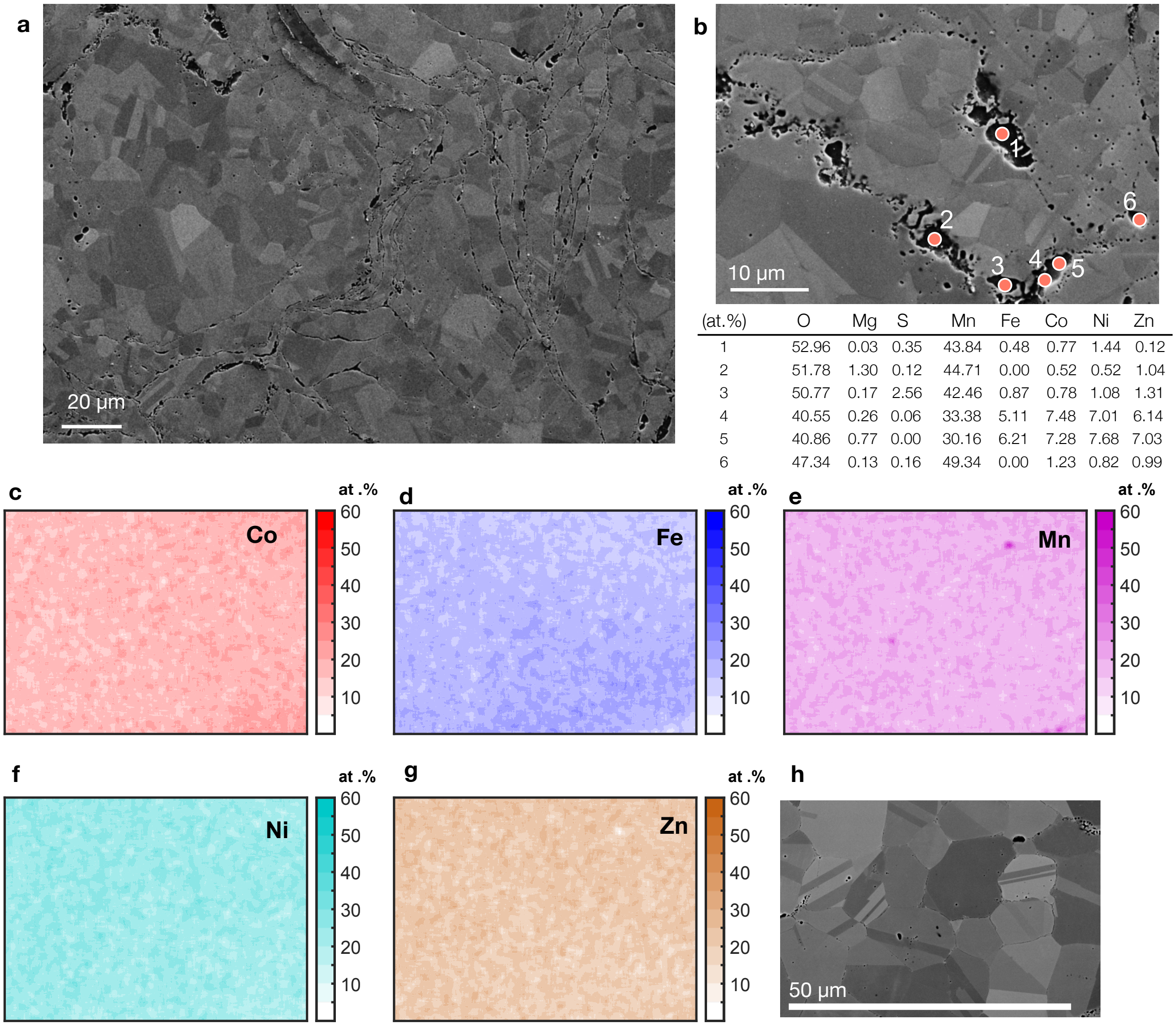

(at.%)	O	Mg	S	Mn	Fe	Co	Ni	Zn
1	52.96	0.03	0.35	43.84	0.48	0.77	1.44	0.12
2	51.78	1.30	0.12	44.71	0.00	0.52	0.52	1.04
3	50.77	0.17	2.56	42.46	0.87	0.78	1.08	1.31
4	40.55	0.26	0.06	33.38	5.11	7.48	7.01	6.14
5	40.86	0.77	0.00	30.16	6.21	7.28	7.68	7.03
6	47.34	0.13	0.16	49.34	0.00	1.23	0.82	0.99

Supplementary Fig. 9. **a** Low magnification (SE) SEM micrograph of CoFeMnNiZn. The single phase FCC grains are decorated by oxides. **b** EDX measurements for a selected number of oxide particles. **c-g** EDX mapping of the FCC matrix of region **h**.

SUPPLEMENTARY REFERENCES

[1] X. Yang and Y. Zhang, Prediction of high-entropy stabilized solid-solution in multi-component alloys, Mater. Chem. Phys. **132**, 233 (2012).

[2] S. Guo, Q. Hu, C. Ng, and C. Liu, More than entropy in high-entropy alloys: Forming solid solutions or amorphous phase, Intermetallics **41**, 96 (2013).

[3] Z. Wang, Y. Huang, Y. Yang, J. Wang, and C. Liu, Atomic-size effect and solid solubility of multicomponent alloys, Scr. Mater. **94**, 28 (2015).

[4] A. K. Singh, N. Kumar, A. Dwivedi, and A. Subramaniam, A geometrical parameter for the formation of disordered solid solutions in multi-component alloys, Intermetallics **53**, 112 (2014).

[5] M. C. Troparevsky, J. R. Morris, P. R. C. Kent, A. R. Lupini, and G. M. Stocks, Criteria for predicting the formation of single-phase high-entropy alloys, Phys. Rev. X **5**, 011041 (2015).

[6] O. Senkov and D. Miracle, A new thermodynamic parameter to predict formation of solid solution or intermetallic phases in high entropy alloys, J. Alloys Compd. **658**, 603 (2016).

[7] S. Guo, C. Ng, J. Lu, and C. T. Liu, Effect of valence electron concentration on stability of fcc or bcc phase in high entropy alloys, J. Appl. Phys. **109**, 103505 (2011).

[8] M. C. Gao, J.-W. Yeh, P. K. Liaw, and Y. Zhang, eds., *High-entropy alloys* (Springer Cham, 2016).

[9] S. Gorsse, M. Nguyen, O. Senkov, and D. Miracle, Database on the mechanical properties of high entropy alloys and complex concentrated alloys, Data in Brief **21**, 2664 (2018).

[10] C. K. H. Borg *et al.*, Expanded dataset of mechanical properties and observed phases of multi-principal element alloys, Sci. Data **7**, 430 (2020).

[11] A. Takeuchi and A. Inoue, Mixing enthalpy of liquid phase calculated by Miedema's scheme and approximated with sub-regular solution model for assessing forming ability of amorphous and glassy alloys, Intermetallics **18**, 1779 (2010).

[12] F. Boer, R. Boom, W. Mattens, A. Miedema, and A. Niessen, *Cohesion in Metals: Transition Metal Alloys*, Cohesion and structure (North-Holland, Amsterdam, Netherlands, 1988).
